# Therapeutic Mask: An Intervention Tool for Psychodrama With Adolescents

**DOI:** 10.3389/fpsyg.2020.588877

**Published:** 2021-01-12

**Authors:** Nuno Pires, José Gregório Rojas, Célia M. D. Sales, Filipa M. Vieira

**Affiliations:** ^1^Higher Institute of Social Work of Porto, Porto, Portugal; ^2^Lusiada Research Center on Social Work and Social Intervention (CLISSIS), Lisbon, Portugal; ^3^Faculty of Psychology and Education Sciences, University of Porto, Porto, Portugal; ^4^Associação Centro Luís de Camões, Funchal, Portugal; ^5^Center for Psychology at University of Porto (CPUP), Porto, Portugal

**Keywords:** psychodrama, masks, adolescents, psychotherapy, mental health

## Abstract

Psychodrama is an effective psychotherapeutic model but interventions with adolescents require age-tailored techniques that maximize engagement and facilitate communication processes. This study describes a novel adaptation of a therapeutic mask technique to psychodrama with adolescents. Over the course of eight group sessions of psychodrama, five adolescents (16 to 18 years-old) created their own mask and explored its therapeutic use. Their experiences were captured at the end of each session with the Helpful Aspects of Therapy (HAT) form, and at the end of the study with the Clinical Change Interview (CCI). Awareness/insight/self-understanding, empowerment and relief were the most significant aspects experienced by the adolescents, along with perceived increase of calmness and world connection, satisfaction in interpersonal communication and better emotional expression and regulation. The mask technique was experienced as a playful and engaging task that facilitated insight and interpersonal communication. Findings provide preliminary evidence on the clinical utility of mask-based psychodrama with adolescents.

## Introduction

Effective psychological intervention during adolescence has been identified as a protective factor throughout the life cycle (Hoag and Burlingame, 1997; Daemi and Vasegh Rahimparvar, 2018). However, therapeutic work with adolescents can be difficult since their quest for autonomy may challenge therapists and hinder therapeutic engagement (Oetzel and Scherer, 2003; Leggett, 2009; Bennett et al., 2017). Moreover, it is also common for teens to have problems in understanding and expressing their thoughts, behaviors, and feelings, which can decrease their communication skills (Leggett, 2009; Bennett et al., 2017). In order to overcome these difficulties, the use of creative therapeutic interventions that match talking and playing have been recommended (Leggett, 2009; Utley and Garza, 2011; Bennett et al., 2017). Creative techniques may aid clients to feel a symbolic safe distance from their therapist, deepening attunement with their inner experiences (ideas and emotions) and facilitate its expression (Leggett, 2009; Utley and Garza, 2011; Bennett et al., 2017). This paper aims to explore the views of adolescents regarding the integration of a mask technique in the therapeutic process of a psychodrama group.

Psychodrama is a group psychotherapy based on action techniques (e.g., role reversal, role-playing, sculptures, doubling, and sociometry), and enhancement of spontaneity and creativity (Cruz et al., 2018; Yaniv, 2018). Through dramatic action, clients can explore and express their internal experiences (e.g., feelings, thoughts), whether past, present or future, exteriorize their problems and simulate life realities, searching for possible solutions for the existing challenges in their lives (Orkibi et al., 2017; Yaniv, 2018; Orkibi and Feniger-Schaal, 2019; Şimşek et al., 2019). In this safe environment, group members can practice the existing roles in different ways or experience new ones, gain insights about themselves or others, and develop life skills (Orkibi et al., 2017; Yaniv, 2018; Orkibi and Feniger-Schaal, 2019). In other words, group members can achieve new perspectives and ways of acting, concerning their problems, and life in general. Psychodrama is a good therapeutic model for working with adolescents, addressing important dimensions for identity construction, such as socialization, feelings of belonging to a group of peers and psychosocial development (Moreno, 1946, cited in Cossa, 2003; Castro and Almeida, 2017; Orkibi et al., 2017).

In order to facilitate the client’s communication, psychodrama can use intermediate objects such as puppets, dolls, masks, among others (Rojas-Bermúdez, 2017). Our work explores the use of the mask as an intermediate object. The earliest reference of the use of masks with therapeutic purposes goes back to 1950s. In an exploratory study, Pollaczek and Homefield (1954, cited in Brigham, 1970 and Janzing, 1998) used masks with a group of children, between 8 and 11 years old, with speech problems. Authors noticed that in sessions, when children performed some role wearing the mask, they reduced their difficulties in maintaining verbal communication. Since then, masks have been applied in therapeutic contexts, using different methodologies such as the use of make-up (Breitenbach, 1979, 1987 and Petzold, 1975, cited in Janzing, 1998) or construction with clay (Sheleen, 1978, 1983, cited in Janzing, 1998), and with different clinical populations, such as bulimic women (Hinz and Ragsdell, 1990), victims of sexual abuse (Trepal-Wollenzier and Wester, 2002), and psychotic patients (Cichocki et al., 2016). According to Landy (1985), the use of masks can facilitate specific therapeutic tasks, namely: when a client needs to represent two sides of a conflict or dilemma; to express his or her identity in a group; to explore dreams and imagery; or to express a social role.

In the late 1960s, Rojas-Bermúdez introduced masks in psychodrama, through his work with chronic psychotic patients. He noticed that psychotics had difficulties in understanding human natural communication, translated by an inability to decode non-verbal signs in others. For the author, the usage of masks would eliminate non-verbal cues and the complexity of facial expressions. Through masks, he observed improvements in patients’ internal and external communication (Rojas-Bermúdez and Moyano, 2012; Rojas-Bermúdez, 2017). Other therapists have observed that using masks as a therapeutic tool helped clients engaging with internal contents, promoted self-knowledge, and facilitated self-disclosure without the pressure created by the direct contact of the face with the social mirror (Brigham, 1970; Janzing, 1998; Rojas-Bermúdez and Moyano, 2012; Castro and Almeida, 2017; Rojas-Bermúdez, 2017). Masks also allowed clients to gain distance from their problems, gaining space to become more reflective (West et al., 2001). Moreover, observing others acting with masks can create a sense of alienation, separating the masked from the observer. This effect can help observers to go deeper in their inner experiences, moving from the message and meaning of what they observe to discover their own meanings (Roy, 2016). Also, in therapeutic work with families, masks provided a sense of anonymity that helped family members to expose their thoughts and feelings more freely without the pressure of responsibility for their behaviors, or the fear of other’s reactions (Baptiste, 1989). At the same time, masks facilitated processes of feedback because the covered face filtered negative impacts of non-verbal communication. Moreover, masks introduced a lively and relaxed environment, and stimulated family members’ cooperation during the elaboration of the masks (Baptiste, 1989; Trepal-Wollenzier and Wester, 2002; Gerity and Bear, 2007).

From the above, there is evidence that the mask technique facilitates important developmental tasks of adolescence, and could serve as a useful therapeutic tool in psychodrama with this population: it facilitates access to internal contents and promotes self-knowledge; it helps go deeper in one’s emotional experiences, to reach insights about oneself and life in general, to communicate clearly, promoting the emergence of a new self; it combines verbal and non-verbal expression of the inner world and behaviors, as suggested for therapeutic work with adolescents (Castro and Almeida, 2017; Daemi and Vasegh Rahimparvar, 2018; Şimşek et al., 2019). However, to our knowledge, the integration of masks in psychodrama processes with adolescents have never been empirically studied. The aim of this study was to understand the adolescents’ experiences on the use of the mask technique as a therapeutic aid in psychodrama. We expected to gain a detailed and richer understanding of the potential of this tool and contribute positively to fruitful psychotherapeutic work with adolescents.

## Materials and Methods

### Study Setting and Participants

The study was conducted using a naturalistic psychodrama group with adolescents, at the Outpatient Center Clinic of the Faculty of Psychology and Education Sciences, University of Porto (FPCEUP). Informed consent was obtained from all participants, but research-based ethical approval was not required, as the group (and evaluation) reflected usual clinical practice within the service. However, compliance with the ethical and deontological principles that were approved by the Ethics Committee of the University of Porto, was safeguarded.

Five adolescents participated in the study, two females and three males, with ages ranging from 16 to 18 years old (*M* = 16.6; *SD* = 0.8). Three participants attended high school and the other two middle school. All participants had been in individual psychotherapy within the previous 12 months. The most reported reason why these clients were referred to psychodrama therapy was anxiety problems, specifically translated in interpersonal relationships difficulties. This was an open format group. In their current therapy the mean duration of treatment was one and a half year, with durations ranging from 2 months to 4 years.

### Mask Technique

In this article, we describe a powerful therapeutic work with masks in a psychodrama group of adolescents. We adapted the brief series of mask technique developed by Rojas-Bermúdez and Moyano (2012); Rojas-Bermúdez (2017), which consists in painting a series of four masks on cardboard. The images and contents revealed symbolically by the masks are first characterized and signified through the filling of a list of attribution of characteristics for each mask (with title/name of the mask; it’s age; in which situation the mask could be used; most and least liked one; most and least intimate/superficial one) and then explored, with different psychodrama techniques (Rojas-Bermúdez and Moyano, 2012; Rojas-Bermúdez, 2017). This technique has the potential of facilitating an intimate encounter of each client with him/herself, allowing the experimentation of differentiated roles (both creator and observer of the creation) and listen the views of the group regarding their mask. Also, the technique can facilitate access, interpretation, evaluation and (re)construction of meanings regarding internal contents represented symbolically in the mask; and how those contents dialogue with individual’s day-to-day life and the relationship he/she/they establish with itself, the group and its therapeutic agents.

The application of the mask’s technique took place during eight weekly 90 min sessions. The session’s structure followed the psychodrama theoretical model: warm-up, enactment, and sharing. The goal of the warm-up phase is to prepare the group for therapeutic work. In the action/enactment phase the group enacts real or imagined life events. The sharing phase consists in a group discussion about the events, feelings and emotions that emerged in the action/enactment phase. Of this block of eight sessions, the first two sessions were structured with a focus in diaphragmatic breathing exercises and body/sensorial exploration. In the subsequent three sessions, therapeutic work involved the preparation of the materials necessary for the mask-making process and the creation of the masks themselves. In the last three sessions, the masks previously created were actively introduced in therapeutic work. Specific interventions included characteristics attribution to each mask, role-playing of different life situations using the mask and dramatic games (e.g., create a psychodramatic form of presentation using their masks, create different characters while embodying the different masks). These eight sessions were considered as a full spectrum of what we consider as the application of the adapted form of Rojas-Bermudez’s proposal of the series of masks technique. All sessions took place in a wide room with chairs, cloths, and other materials available, namely cardboard, brushes, finger painting, mirror, pencils, scissors. In all sessions were present the group director, auxiliary ego, and the group members.

### Measures

#### Helpful Aspects of Therapy (HAT)

The HAT (Llewelyn et al., 1988; Sales et al., 2007b) is a post-session self-report questionnaire that addresses clients’ perceptions of significant therapy events (SE). Clients are asked to identify and describe, in their own words, the most helpful and hindering events at the end of each session. By event we mean something that happened, something that was said or done, or a specific activity. The HAT includes questions such as “Of the events which occurred in this session, which one do you feel was the most helpful or important for you?”; “What made this event helpful/important and what you got out of it?”; “Did anything happen during the session which might have been hindering?”

#### Clinical Change Interview (CCI)

The CCI (Elliott et al., 2001; Sales et al., 2007a) is a semi-structured interview, with four to eight open-ended questions, that seeks to explore the changes that clients’ might have noticed since the beginning of therapy, what clients’ attribute these changes to, and helpful and hindering aspects of therapy. For each change identified, the client is asked to indicate how surprising the change was (in a 5 point scale from 1- expected to 5 – surprised), if the change would have occurred without therapy (5 point scale from 1- unlikely to 5 – likely), and how important the change was (5 point scale from 1- not at all to 5 – extremely important). The CCI can be administered at the end of the therapeutic process and at regular intervals throughout therapy. The interview lasts, in average, between 30 and 45 min.

### Procedure

#### Data Collection

The study followed the group weekly between June and August of 2017. The psychodrama group was led by an experienced and trained director (NP). During the research, one member of the research team (JGR), who is a therapist in training in psychodrama, was part of the therapeutic team as an auxiliary ego. On a weekly basis, the HAT form was sent via e-mail to the clients after each session. At the end of the data collection process for the study, each adolescent was interviewed by JGR (CCI). The interviews were audio recorded and transcribed.

#### Data Analysis

The content of the qualitative data provided by the HAT and CCI were analyzed by two independent raters in order to derive meaning and identify recurring conceptual patterns of experience, with the aim of organizing the information in different categories (Elliott and Timulak, 2005). A thematic analysis was conducted (Braun and Clarke, 2006). For the HAT, a deductive approach was used. The SE were categorized from the adaptation of a frame of categories of significant events in psychotherapy, available in the literature (Elliott, 1985, 2010; Timulak, 2007, 2010; Castonguay, 2011; Richards and Timulak, 2012; Cruz, 2014; Vieira, 2014; Cruz et al., 2016). If a client response did not match clearly with the original categories, the events was categorized in a new one. This happened specifically with events related to the mask technique (mask technique, sensorial exploration exercises, diaphragmatic breathing exercises).

The SE were organized in two conceptual domains – Impact (effects/results that the session had on the participant) and Action (SE that describe specific situations/exercises that occurred during the sessions). Each domain was organized in thematic categories. The codification in each category was not mutually exclusive. The kappa coefficient for HAT was 0.74. Frequencies of responses in each category were analyzed. For the CCI an inductive and data-based approach was used. The transcripts were closely examined to identify common themes – topics, ideas and patterns of meaning that come up repeatedly. Clients identified a broad diversity of changes that were organized into six thematic categories. A kappa coefficient of 0.83 was found.

## Results

### Significant Events

Over the course of the eight psychodrama sessions, adolescents reported 96 helpful SE, and three hindering SE in their HAT forms. Tables 1, 2 display a brief description of the categories found in each two domains, as well as some illustrative quotes.

**TABLE 1:** HAT- description and illustrative quotes of SE – Impact Domain.

	Description	Illustrative quotes
**Helpful events (*n* = 96)**		
Insight/Self-awareness/Self-knowledge (*n* = 30)	The individual becomes more aware of his/her feelings, cognitions and behaviors Developing a clearer understanding of himself/herself, others, and situations Development of a new perspective on the personal reality	“I noticed that I had more “similarities” with my mask than what I thought” (p4s6m71) “The activity made me think a bit about the way I face and deal with bullying situations. The tension I accumulated in a simple enactment showed me that there are some aspects I need to work more, and it was important for me to become aware of that.” (p2s8m88)
Empowerment/(*n* = 20)	Development of personal competence to deal with problematic situations Feeling of strength, development, and personal value	“This event helped me because, sometimes, I would suffer with panic attacks and couldn’t control my breathing and it also helped to become calmer, because with a good breath everything is easier” (p3s1m8) “I think I learned that maybe it’s not worth to always be so perfectionist” (p2s5m52d)
Problem Clarification (*n* = 5)	The individual relates a clearer understanding of his/her problems or what he/she wants to change It becomes clear to the individual which goals to achieve, how to handle situations	“It helped me to deal and understand better some aspects of my personality.” (p4s6m72) “The tension I accumulated in a simple enactment showed me there are still some aspects I need to work on. I believe it was important to become aware of that” (p2s8m88)
Relief (*n* = 17)	Client feels less negative: relieved, unburdened, relaxed, less depressed, or hurt; or more positive: relaxed, safe, or confident or hopeful\	“During the session I felt comfortable, calm and focused.” (p2s4m40c) “It’s always good to get to know our body. Doing these sessions relaxes me and I feel good” (p3s4m42)
Group Factors (*n* = 13)	Cohesion feelings, support, safety, and well-being related to the group Feeling understood by the group; relativizing problem by comparison with the other; feelings of group identity, universality, and mutual aid	“I enjoyed the warm-up because it helped me to trust more in others” (p4s5m58) “It helped me to understand how I see myself and how others see me” (p3s6m68) “To realize that in some situations it is easier to work in a group than alone” (p1s3m27a)
Session evaluation (*n* = 11)	Global positive appreciation of the session	“I enjoyed it very much and it was fun and interesting” (p1s3m28) “The session was pleasant, considering I never worked in something more practical in such a relaxed way” (p2s4m40a)
**Hindering events (*n* = 3)**		
Unwanted Thoughts/Feelings (*n* = 3)	Client feels discomfort as a result of being forced or stimulated to confront unpleasant experiences	“I didn’t enjoy the sensorial exploration exercises because I didn’t feel comfortable” (p4s4m46) “I didn’t do the last exercise because I didn’t feel comfortable to do it” (p1s7m76)

*p0s0, participant 0, session 0.*

**TABLE 2:** HAT- description and illustrative quotes of SE – Action Domain.

Action	Description	Illustrative quotes
Enactment – Being an auxiliary-ego (*n* = 1)	Client describes the appropriation of the auxiliary ego role in the enactment phase	“Representing the bully” (p2s8m86)
Final Comments/Others’ sharing (*n* = 3)	Client refers to the comments shared by others (either peers or therapists) at the end of the session	“To listen some comments by the end of the session” (p2s7m78)
Sculpture (*n* = 1)	Symbolic representation of the way protagonist perceives an aspect of his/her life or self	“It was very positive, although I didn’t do the exercise because I didn’t feel comfortable enough to do it” (p1s7m76)
Games (*n* = 10)	A game with specific objectives and specific rules, proposed by the director	“In the last session we “played” to the “sages,” which was interesting because we had to be keen and creative” (p3s3m31)
Mask technique (*n* = 16)	Client describes the process of preparing creating and caracterizing the set of masks (according to the *serie de* máscaras technique), as well as the usage of self-masks as an interintermidiary object in the enactment phase or games	“In this session what helped me was to give different characteristics to the masks we had made on the previous session” (p1s6m61) “Revisiting the masks.” (p4s6m70)
Sensorial Exploration exercises (*n* = 8)	Client describes moments of the sensorial exploration exercises	“To become aware of how my hands were” (p2s4m38)
Diaphragmatic Breathing exercises (*n* = 5)	Client refers the diaphragmatic breathing exercises and their impacts	“To become aware of my breath and what I felt by breathing” (p2s1m4)

*p0s0, participant 0, session 0.*

The most frequent helpful SE reported by the adolescents during these sessions were related to an increased awareness and self-knowledge (31.3% of the helpful SE). As some of the participants put it, “It helped because I could see how I see myself and how others see me,” “It allowed me to reflect a bit more on how I interact (or try to interact) with others,” or “I realized that I feel the need to talk to people.” During these eight sessions, adolescents also experienced empowerment (20.8% of the helpful SE) and feelings of calm, relief, and well-being (17.7% of the helpful SE): “Talking about bullying and remembering how I handled it helped me create a new interpretation,” “This event helped me because, sometimes, I suffered from anxiety attacks and I couldn’t control my breathing, and it also helped me to be calmer because with a good breath everything is easier.” Although to a less extent (3% of the total SE) adolescents also reported hindering SE, mostly related to inhibition and discomfort during the early sessions of the use of masks: “Personally I did not like the sensorial exploration exercises, as I did not feel comfortable” and “I didn’t do the last exercise because I didn’t feel comfortable doing it.”

The mask technique (36% of the total SE) and the psychodramatic games (23% of the total SE) were the actions that adolescents perceived as most significant over the course of these eight sessions (Table 2). Both during the first part of the games, as well as when exposing the masks to others, participants stressed “It also allowed me to feel even more comfortable with the group” or “I found it interesting because, in the handwork we had to interact with each other and communicate with the group to work properly.”

### Perceived Changes

In CCI, adolescents identified a total of 16 changes that were retrospectively attributed to the psychodrama group integrating the masks (see Table 3). These changes were thematically organized into six categories. The most frequent changes were a greater experience of calm and relax and increasing interpersonal communication skills (both reported by four adolescents): “It was a good feeling, to relax without feeling that I was being observed. I ended up taking this a bit with me,” “I can feel calmer, more comfortable to speak,” “I was able to communicate a bit more, inside and outsider the group” or “At school, I spoke with some of my classmates and a teacher. And I have communicated more with my family.” Three adolescents also stressed improvements in emotion regulation and emotion expression. Two adolescents highlight a better understanding and acceptance of past experiences, and a more involvement on daily routines and, in one case, a greater comfort with physical touch: “This way it was easier, it gave me more ways to speak, I didn’t need to do any gestures,” “I’m able to look to certain things I’ve been through, but that now I know how to act,” “I’m feeling freer because I don’t think so much in what others are saying” or “I used to not like people to touch me or hug me. I feel that here, at therapy, with the exercises, it’s something that has been helping me.”

**TABLE 3:** Changes identified in the CCI.

Changes	*n* (%)	Illustrative quotes
Greater experience of calm and attention to the other	4 (25.0)	“I can feel calmer, more comfortable to speak” “To be more focused, and not closing myself so much in my thoughts”
Improvements in interpersonal communication (with adults and peers)	4 (25.0)	“I felt more comfortable with some people (of the group) which also made me feel more confident in general” “I was able to communicate a bit more, inside and outsider the group”
Improvements in expression and emotional regulation	3 (18.8)	“This way it was easier, it gave me more ways to speak, I didn’t need to do any gestures. My mask already represented everything” “Now I will even cry in the street sometimes, if I see something, I get emotional and start crying”
Understanding and acceptance of past experiences	2 (12.5)	“I’m able to look to certain things I’ve been through, but that now I know how to act” “It helped me to better understand the part of not paying attention. I already didn’t pay, but that part (of the session) helped me even more”
More involvement in day-to-day tasks	2 (12.5)	“Usually I do things very polished, exactly how others are doing it. It was nice to not follow that process. It was a good feeling to be able to do it (the mask task) relaxed, without feeling I was being judged, because often I have that perception although it doesn’t exist” “Now I pretend I’m not paying any attention, (and) I’m feeling freer because I don’t think so much in what others are saying”
More comfortable with the touch	1 (6.2)	“I used to not like people to touch me or hug me (…) I feel that here, at therapy, with the exercises, it’s something that has been helping me. And with this touch “thing” I realize I’m progressing”

When asked about how surprising or unexpected these changes were, adolescents report that 63% of the changes were somewhat or totally surprising. And 81% of the changes would not have happened without the psychodrama integrating the mask technique. About the importance attributed, 75% were considered extremely important changes.

## Discussion

The current study sought to investigate the views of a group of five adolescents enrolled in a psychodrama treatment enriched with the mask technique. It contributes to new reflections about psychodrama work with adolescents, focusing the usage of masks as a therapeutic tool. The findings suggest that the insight/self-awareness, self-overcoming, self-empowerment, the experience of well-being/calm/relief/focus, and the group factors were the most significant experiences within the therapeutic process. Adolescents reported masks technique (construction of masks and characteristics assignment to each one), and their usage in psychodramatic games and enactment, as the most helpful for reaching new insight/self-awareness/self-knowledge about themselves.

The positive impacts on insight and self-awareness are in line with previous psychodrama-based therapeutic work with adolescents (Cossa, 2003; Orkibi et al., 2017; Şimşek et al., 2019). Psychodrama appears as a mean of reaching this self-knowledge for adolescents, providing strength and security in the relationship between peers (Castro and Almeida, 2017; Fontoura et al., 2020). These results are also congruent with studies about the impact of psychodramatic intervention with adults (McVea et al., 2011; Cruz, 2014; Vieira, 2014) and with a systematic review about SE (Timulak, 2010) which revealed that insight events are the most important aspect of this therapeutic model.

Because psychodrama uses action as a base, it provides adolescents the opportunity to enact and realize their difficulties, in addition to providing communication tools with other group members. As expected, mask-based psychodrama helped adolescents to go deeper in their inner experiences, reaching new perspectives about themselves or others. Adolescents emphasized the development of a clearer understanding of themselves and reality, and an increase of personal skills to deal with problematic situations. These data are in line with Timulak and McElvaney (2013) that observed two types of insight events, one that potentiates clients’ perception of their problems, and other that enlights what they want to change.

The experiences of self-overcoming/empowerment, well-being/calm/relief/focus, which are related to internal work, were associated to tasks such as relaxation and diaphragmatic breathing, mask construction and enactment with mask. Once again, these data reinforce the role that the mask technique played in the therapeutic process. They underline an empowering process based on emotions experienced in body and translated cognitively, which can develop consciousness of personal resources, and promote a sense of capability to deal with problematic issues (Timulak and Elliott, 2003).

The core aspects that lead adolescents to identify Group Factors/Interpersonal Relations as helpful could also be related to mask technique. The tasks associated to the mask technique promoted an attunement within group members, which helped to create a new relational experience and connection. This is consistent with Baptiste’s (1989) experience of emerging cooperative stance between family members during the creation of the masks. These results highlight an important contribution of this technique to promote interpersonal skills, which was the principal motive of reference to psychodrama.

The hindering events emerged from techniques that appealed the use of the body, like sensorial exploration and sculpture construction. Interestingly, despite the provoked discomfort, delivered techniques based on body sensations or expression seemed to have a noteworthy impact, by encouraging adolescents’ attendance to their feelings, allowing them to gain a better understanding of what they do not like, or what they need. It is also important to note that felt difficulties may be due to personal characteristics of the adolescents, such as aggressiveness, absence of adaptive emotional regulation strategies, and communication problems. Notwithstanding the scarcity of hindering events, they should be considered, because they can cause ruptures in therapy, decreasing clients’ engagement in treatment (Safran et al., 2011). In this case, it seems important that therapists consider with caution the use of the masks in exercises that appeal the use of the body, always considering the adolescent’s characteristics in order to avoid exercises that can be harmful.

There is a clear association between the helpful events experienced during these eight sessions and the changes presented in the CCI. Adolescents declared as major changes a greater experience of calm and involvement with the world in an attentive and peaceful manner, greater satisfaction in the establishment of interpersonal communication and in relationships with peers and adults, as well as changes regarding emotional expression and emotional regulation. We highlight an adolescent that stated, “With the mask, nobody could see if I was smiling, if I was crying, and I could show my normal face and people wouldn’t see.” That statement goes in line with literature that predicts that wearing the mask allows the communication of emotional states and internal contents without the pressure created by the direct face-to-face contact, hence increasing the engagement in enactment (Brigham, 1970; Janzing, 1998; Rojas-Bermúdez and Moyano, 2012; Rojas-Bermúdez, 2017). As if being behind a mask promoted a feeling of safeness to go deeper to the “real person,” and to create new evaluations of oneself and of others (Baptiste, 1989; Leggett, 2009). Creative play with masks promotes the contact with others and the emergence of new perspectives about reality (Leggett, 2009; Utley and Garza, 2011; Bennett et al., 2017), therefore allowing to see or discover the real person behind the mask.

In sum, the Rojas-Bermúdez mask technique used as an intermediate object in psychodrama with adolescents seems to facilitate psychological reactions that promote access to insight, self-knowledge and a better understanding of the internal and external reality, which are fundamental aspects for personality development, a core aspect of human development throughout the life cycle. It is an age-appropriate technique for adolescents that complies with recommendations for creative techniques that join talking and playing, and reinforces clients’ appreciation of the experiential nature of psychodrama (Orkibi et al., 2017). Mask-based psychodrama creates a therapeutic atmosphere that favors the establishment of interpersonal relationships, the solution encounter to intra and interpersonal problems as well as learning to deal with adolescents’ typical challenges (Castro and Almeida, 2017; Orkibi et al., 2017; Daemi and Vasegh Rahimparvar, 2018). Our results provide preliminary evidence of masks as clinically relevant and appreciated therapeutic tools that can enrich psychodramatic interventions with adolescents.

This study should be interpreted considering its limitations. Data comes from a small sample size and clients’ perceptions are idiosyncratic. Therefore, results are unrepresentative to general population and cannot be generalized. Despite the data analysis was made by two researchers, researcher bias can be present, given the team allegiance to psychodramatic approaches. Although the reinforcing results of masks usage in therapy, we cannot clearly establish whether the therapeutic benefits were specifically due to this technique, because its introduction was carried out in an ongoing therapeutic process. Nonetheless, the findings of the current study point to the potential of masks as a useful tool to enrich psychodrama processes with adolescents.

## Data Availability Statement

The raw data supporting the conclusion of this article will be made available by the authors, without undue reservation.

## Ethics Statement

Ethical review and approval was not required for the study on human participants in accordance with the Local Legislation and Institutional Requirements. Written informed consent to participate in this study was provided by the participants’ legal guardian/next of kin.

## Author Contributions

FV and CS supervised the research work. NP, JR, and FV had full access to all the data in the study and took responsibility for the integrity of the data and the accuracy of the data analysis. JR, NP, CS, and FV contributed to the study design and wrote the manuscript. JR and NP contributed to the data collection. JR, CS, and FV contributed to the data analysis. All authors reviewed and approved the final version of the manuscript.

## Conflict of Interest

The authors declare that the research was conducted in the absence of any commercial or financial relationships that could be construed as a potential conflict of interest.
